# Association between the Perceived Household Financial Decline Due to COVID-19 and Smartphone Dependency among Korean Adolescents

**DOI:** 10.3390/ijerph19063303

**Published:** 2022-03-11

**Authors:** Yun Hwa Jung, Soo Young Kim, Sung-In Jang, Eun-Cheol Park, Jaeyong Shin, Junghwan Suh

**Affiliations:** 1 Department of Public Health, Graduate School, Yonsei University, Seoul 03722, Korea; yunhwa@yuhs.ac (Y.H.J.); skim21@yuhs.ac (S.Y.K.); 2 Institute of Health Services Research, Yonsei University, Seoul 03722, Korea; jangsi@yuhs.ac (S.-I.J.); ecpark@yuhs.ac (E.-C.P.); 3 Department of Preventive Medicine, Yonsei University College of Medicine, Seoul 03722, Korea; 4 Department of Pediatrics, Severance Children’s Hospital, Yonsei University College of Medicine, Seoul 03722, Korea

**Keywords:** COVID-19, income, poverty, smartphone, addictive behavior, adolescent

## Abstract

This cross-sectional study identified the association between COVID-19-related perceived household financial decline and smartphone dependency among adolescents in South Korea. Data from the 2020 Youth Risk Behavior Survey of Korea was used and 54,809 middle and high school students were included. COVID-19-related perceived household financial decline was categorized as no financial decline, mild, moderate, and severe. Smartphone dependency was calculated by 10 questions and was largely categorized as yes and no, and as normal, low, and high (prevalence rate: 25.0%). Binary and multinomial regression analyses were performed to analyze the association. The more severe the financial decline, the more pronounced the risk of high-risk smartphone dependency (mild financial decline: odds ratio (OR) 1.11, 95% CI 0.96–1.28; moderate: OR 1.22, 95% CI 1.04–1.43; severe: OR 2.56, 95% CI 2.06–3.17). Poor family relationships (OR 1.06, 95% CI 1.03–1.10) and severe social conflict (OR 2.99, 95% CI 2.50–3.58) were also related to smartphone dependency. The ORs were 2.63 with more than three bathrooms and 1.63 with their own bedroom. Smartphone dependency among adolescents is closely related to COVID-19-related perceived household financial decline. As smartphone dependency relates to complicated psychological issues, further evaluation is necessary, especially for vulnerable adolescents.

## 1. Introduction

South Korea’s smartphone penetration rate was 95.9% among middle school students and 95.2% among high school students in 2018 [1]. The proportion of smartphone dependency risk in Korea has been increasing every year, and in 2020 81.9% of Koreans considered smartphone dependency was a serious societal concern [2]. Among Korean adolescents, the proportion of smartphone dependency risk has been rising progressively, and the rate reached 35.8% in 2020 [2]. In previous studies, the prevalence of smartphone problems was 10% among 11–18-year-olds in England, 20% among 13–20-year-olds in Spain, 22.8% among 12–15-year-olds in China, and 62.6% among 13–18-year-olds in the Philippines [3,4,5,6]. These differences may be attributed to variations in age groups, health literacy, and smartphone questionnaire composition.

Smartphone dependency can simply be defined as the state of excessive smartphone use. In other words, smartphone dependence is a state in which the use of smartphones in daily life is prominent and the use of smartphones is not controlled. Among Korean adolescents who are at risk of smartphone dependency, the most serious risk factor was a failure to control smartphone use [2]. Problematic outcomes mean continuously experiencing negative physical, psychological and social consequences of using a smartphone. Smartphone dependency has been associated with physical problems including musculoskeletal disorders, migraines, pain in the wrists and neck, blurred vision, and poor hand function [7,8,9,10,11]. It could also cause psychosocial disorders, including depression, social anxiety, impulsivity, and sleeping disorders [12,13]. In particular, frequent smartphone use could lead to instability and impulsivity, loss of self-control, and withdrawal symptoms in adolescents [14]. Therefore, it is important to understand and prevent smartphone dependency in adolescents, since this can contribute to preventing the consequences of physical, psychological and social problems in this group.

COVID-19 emerged as a novel coronavirus pneumonia in December 2019 and was declared a pandemic by the World Health Organization (WHO) in March 2020. By June 2021, the cumulative number of confirmed cases in Korea was 156,961, accounting for about 0.30% of the total population [15,16]. It is estimated that Korea lost 460,000 jobs in the past year due to the coronavirus crisis, and its annual GDP growth rate fell by 3.7% [17].

Pandemic shocks were experienced across the affected countries, causing economic havoc. COVID-19 has caused unprecedented conditions for millions of families around the world, and individuals suffered tremendous economic and psychological distress [18]. In previous studies, unemployment induced high levels of depression, irritability, and anxiety, and showed a low response to cortisol-related stressors [19]. Parents’ low mood lowered their children’s psychological well-being [20]. COVID-19 can cause increased stress for parents and children, along with loss of income, the burden of childcare, and illness.

Adolescents perceive their family’s income situation relatively subjectively. This is because they do not usually act as economic agents like their parents, or they do not have as much economic knowledge as ordinary adults. For adolescents, subjective material well-being is generally considered important, since its lack in adolescence is a risk factor for adolescents’ social development and mental health. Adolescents are more sensitive to social acceptance or rejection of their peers than are children or adults, and they tend to seek strong bonds with their peers [21]. The social brain develops throughout adolescence. Early in development, the role of caregivers such as parents is important, and after development the peer environment becomes additionally important [22]. Therefore, the lack of subjective material well-being from parents during adolescence can interfere with peer bonds, and there is a risk of unhealthy mental health. 75% of adults with mental health problems experienced symptoms before the age of 24 [23]. Related evidence included problems with peer relationships, bullying, and depression.

Adolescents use the internet, social media and smartphones to connect with their peers. Continuous communication using digital technology without time and space limitations creates a high risk of causing psychological problems. In a previous study, internet addiction in Italy was strongly associated with emotional regulation and dysregulation [24]. Problematic smartphone use can lead to fear of missing out (FoMO) and increase dependence and addiction. FoMO is an individual fear of missing the social flow or of being marginalized. On the other hand, a consistent view has not been formed regarding health issues caused by smartphones related to income. In previous studies, health problems related to smartphone addiction were more prominent among low-income class in Saudi Arabia and low socioeconomic status in Taiwan [25,26]. In contrast, Spain and Malaysia had intensive cellphone use in more affluent families [6,27].

Smartphones are a necessity for modern people, for communication and entertainment, especially for adolescents, for whom peer bonds are more important than for any other age group. Although perceived household financial status is beyond the control of adolescents, it is a factor that greatly affects their present and future mental health. Therefore, investigating the association of smartphone dependency according to perceived household financial decline in adolescence would affect the mental health of adolescents, especially during the historic pandemic COVID-19. Therefore, this study aimed to understand the association between smartphone dependency among adolescents and the financial threat to their families due to COVID-19. We investigated items in the smartphone dependency questionnaire and assessed which of these were more affected by COVID-19-related familial financial issues, with a focus on adolescents. We also studied the degree of severity of adolescent smartphone dependency based on the changing financial situations of their families. In this study, we hypothesized that greater perceived household financial decline is associated with severe smartphone dependency in adolescents.

## 2. Materials and Methods

### 2.1. Data

This study used research data from the 16th Korea Youth Risk Behavior Survey (KYRBS), conducted in 2020. The KYRBS is a representative, anonymous, self-reported online survey of Korean adolescents conducted by the Korea Disease Control and Prevention Agency (KDCA). Since 2005, the KDCA has been conducting annual, nationwide surveys with middle and high school students. The survey for our study was conducted from August to November 2020. This study was stratified by 39 regions and school-types in variables and by school and class in the samples. There were 103 questions covering topics including socioeconomic status, health behavior, health education, and health screening. The research data were de-identified, and anonymity and confidentiality were guaranteed. This study has been approved for exemption from deliberation by the Institutional Review Board of Yonsei University Health System, Severance Hospital in Korea and the approval number was 4-2021-1050.

### 2.2. Participants

This study attempted to survey 57,925 students nationwide. The response rate was 94.9% (54,948/57,925) in 400 middle schools and 400 high schools. Among the respondents, 139 individuals of uncertain age were excluded as missing data, likely because of the excessive workload of teachers in charge of this survey and the unavailability of computer labs during COVID-19. 1275 students were also excluded since they did not measure height, used to calculate the covariate BMI. A total of 53,534 adolescents were finally analyzed in the study, of which 27,687 were boys and 25,847 were girls.

### 2.3. Variables

The independent variable was household financial decline due to COVID-19. which was a subjective perception of adolescents. Levels of financial decline by COVID-19 were categorized as generally “yes” or “no”, and specifically classified as “no financial decline”, “mild”, “moderate” and “severe.”

The dependent variable consisted of the total smartphone dependency scores calculated by the self-reported questionnaire. The questionnaire consisted of 10 questions measuring respondents’ psychosocial difficulties with daily smartphone use. In 2016, the National Information Society Agency developed this questionnaire by integrating the existing Internet scale (K-scale) and smartphone scale (S-scale) into a “smartphone dependency scale”. The questionnaire consists of three items of control failure, three items of salience, and four items of problematic outcomes related to smartphone dependency from 2020 KYRBS. Smartphone dependency is investigated once every 3 years, and only 3 items of problematic outcomes were surveyed in 2017. The item of time spent on smartphones was surveyed in both years. We analyzed the common 4 items of smartphone dependency in the analysis of before and after COVID-19. In the confirmatory factor analysis performed by the National Information Society Agency, the factor loading of all items was good, at 0.5 or higher, and the model fit index was excellent. The overall scale reliability analysis result was also excellent at 0.84 [2]. The smartphone dependency risk group included a low-risk and a high-risk group. As a result of the receiver operating characteristic (ROC) analysis based on the DSM-5′s Internet gaming disorder, the area under curve (AUC) was 0.736, sensitivity 0.943, and specificity 0.529 in the low-risk group; the AUC was 0.629, sensitivity 0.336, and specificity 0.921 in the high-risk group. We focused on the smartphone dependency score to distinguish whether participants could be classified into the smartphone dependency risk group. The scale was categorized as generally around 23 points, specifically 0–22 points (normal), 23–30 points (low-risk group), and 31–40 points (high-risk group).

The covariates were demographics (gender, school grade), socioeconomic factors (household income, financial support, family status, academic level, family affluence), a variable related to the coronavirus disease (COVID-19 infected city), variables related to mental health (perceived stress, sleep duration), a health behavior variable (exercising), and a physical variable (body mass index).

### 2.4. Statistical Analysis

**Figure 1:**
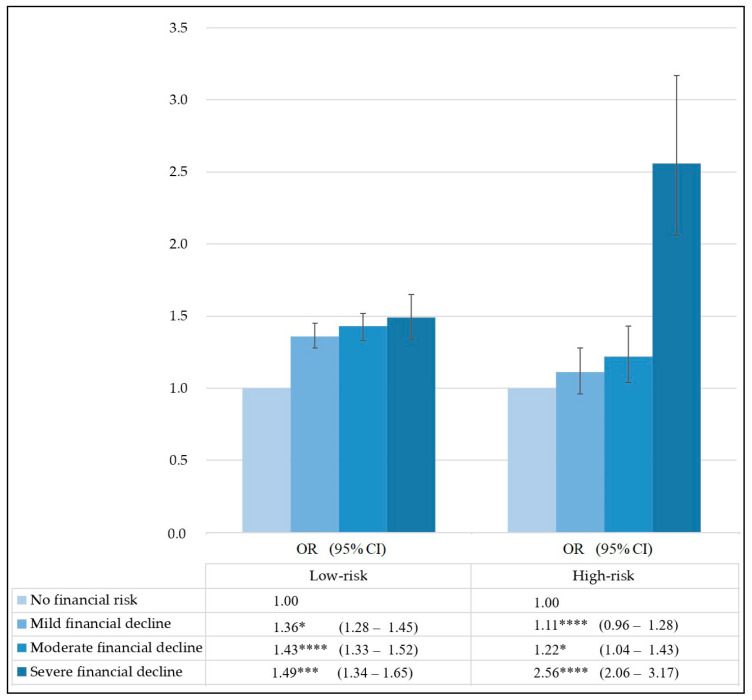
Results of household financial decline due to COVID-19 associated with smartphone dependency (The severity of smartphone dependency on household financial decline due to COVID-19 was analyzed by comparing the low-risk group and the high-risk group. Reference group: No financial decline. *: *p* ≤ 0.05, ***: *p* ≤ 0.001, ****: *p* ≤ 0.0001).

**Figure 2:**
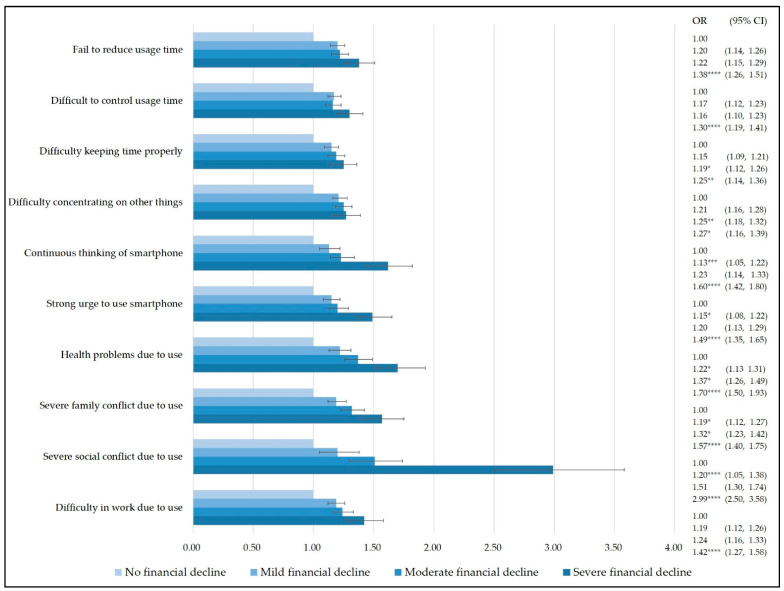
Results of subgroup analysis stratified by smartphone dependency questions (The risk of each item on the smartphone dependency scale was analyzed according to the severity of household financial decline. Reference group: No financial decline. *: *p* ≤ 0.05, **: *p* ≤ 0.01, ***: *p* ≤ 0.001, ****: *p* ≤ 0.0001).

**Figure 3:**
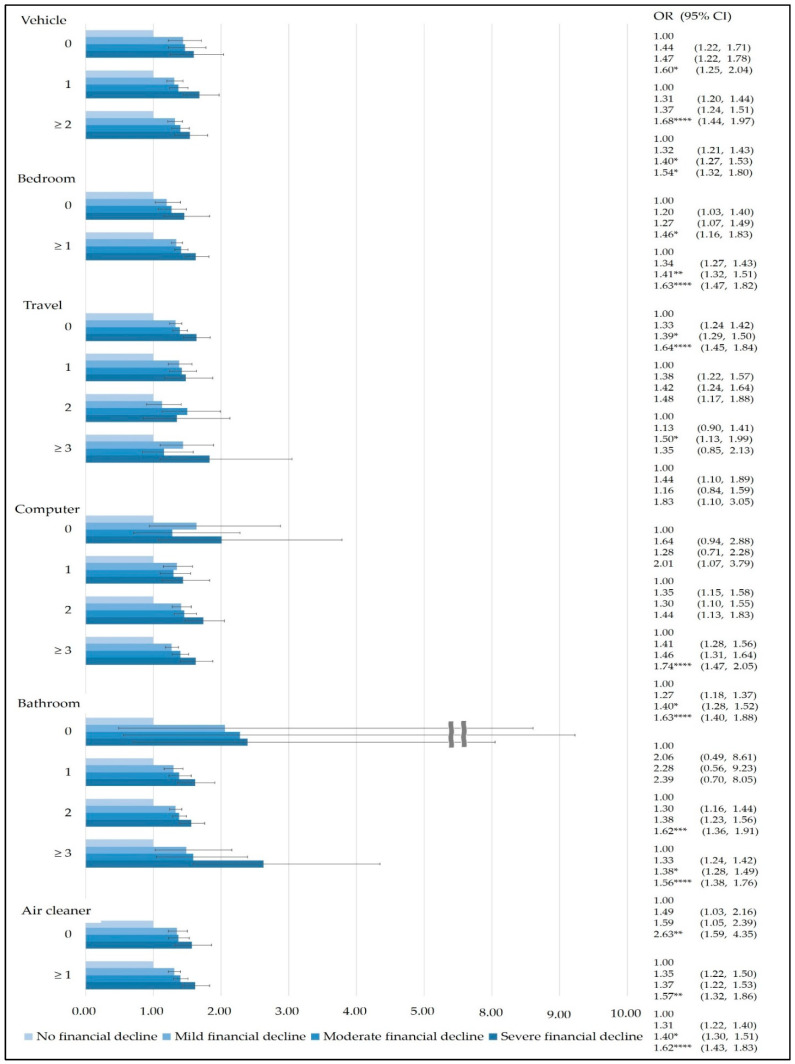
Results of subgroup analysis stratified by family affluence questions (The risk of smartphone dependency due household financial decline caused by COVID-19 was analyzed by each item on the family affluence scale. Reference group: No financial decline. *: *p* ≤ 0.05, **: *p* ≤ 0.01, ***: *p* ≤ 0.001, ****: *p* ≤ 0.0001).

To evaluate the association between household financial decline due to COVID-19 and smartphone dependency, we reported on the general characteristics of the study population as a previous analysis. In Table 1, the prevalence of smartphone dependency was distinguished in each characteristic. As the main analysis, we conducted binary logistic regression using PROC SURVEYLOGISTIC with weight, cluster, and strata for analysis. Results included odds ratios (ORs) and 95% confidence intervals (CIs). There was no multicollinearity using the variance inflation factor (VIF) in any variables. To elaborate the dose-response relationship, multinomial logistic regression was also performed as sensitivity analysis and was used for the analysis of Figure 1, Table 2 and Table 3. Binary logistic regression was used for Figure 2 and Figure 3 analysis. A trend test was conducted to identify the relationship between independent and dependent variables; *p* ≤ 0.05 was statistically significant. Statistical analysis was performed using SAS, version 9.4 (SAS Institute Inc., Cary, NC, USA).

As this analysis was not pre-registered on a publicly available platform, the results should be considered as exploratory.

## 3. Results

The gender ratio among the 53,534 adolescents was similar, with 27,687 boys (51.7%) and 25,847 girls (48.3%). The mean age of students was 15.0 ± 1.7 (Table 1). Regarding the household financial decline due to COVID-19, participants responded as follows: 15,923 “no financial decline” (29.7%); 21,350 “mild financial decline” (39.9%); 13,189 “moderate financial decline” (24.6%); and 3072 “severe financial decline” (5.7%). The overall prevalence of smartphone dependency was 25.0% (13,404/53,534). The prevalence of smartphone dependency increased with household financial decline (no financial decline: 20.0%, mild: 26.3%, moderate: 27.8%, severe: 30.3%).

Adolescents with smartphone dependency indicated gradually increased ORs of household financial decline due to COVID-19 compared to no financial decline (mild financial decline: OR 1.33, 95% CI 1.26–1.41, moderate: OR 1.40, 95% CI 1.32–1.49, severe: OR 1.62, 95% CI 1.47–1.78). Further, significant results in the linear hypotheses testing were also found (*p* for trend <0.0001) (Supplementary Table S1). When household financial decline due to COVID-19 was severe, these individuals began experiencing interpersonal conflicts or problems with daily roles, with weakened control over smartphone use leading to smartphone dependency.

Based on multinomial regression, we investigated the association between the intensity of smartphone dependency and household financial decline. The results revealed that severe financial decline is related to low-risk (OR 1.49, 95% CI 1.34–1.65) and high-risk (OR 2.56, 95% CI 2.06–3.17) dependency, with a statistical increase in trends following increasing risk of dependency (Figure 1).

Further, a subgroup analysis was performed on the high-risk group concerning severe financial decline. Girls and groups with no financial support such as for tuition, lunch, school uniforms, and textbooks from people or institutions were more vulnerable to smartphone dependency than their counterparts (boys and having financial support, respectively) when experiencing worsening household financial decline due to COVID-19 (girls: OR 3.11, 95% CI 2.32–4.16, boys: OR 2.09, 95% CI 1.54–2.83; no financial support, OR 2.56, 95% CI 2.05–3.21, having financial support OR 2.01, 95% CI 1.05–3.86) (Table 2).

Regarding the results of the smartphone dependency questions, the most influential smartphone dependency item associated with household financial decline due to COVID-19 was “severe social conflict due to smartphone use” (mild financial decline: OR 1.20, 95% CI 1.05–1.38; moderate: OR 1.51, 95% CI 1.30–1.74; severe: OR 2.99, 95% CI 2.50–3.58) (Figure 2).

The findings from the family affluence scale questions revealed that adolescents with smartphone dependency whose homes had more than three bathrooms and household financial decline due to COVID-19 was severe (OR 2.63, 95% CI 1.59–4.35). Among adolescents with mild-to-severe household financial decline due to COVID-19, smartphone dependency tended to increase linearly for those with more than one bedroom (mild financial decline: OR 1.34, 95% CI 1.27–1.43; moderate: OR 1.41, 95% CI 1.32–1.51; severe: OR 1.63, 95% CI 1.47–1.82) (Figure 3).

Low-income households in 2020 had higher OR values concerning smartphone use problems than high-income households in 2017. Regarding problems arising from smartphone use in 2020, the OR of family conflict was 1.06 (95% CI 1.03–1.10) and that of difficulty in work was 1.04 (95% CI 1.01–1.07) (Table 3).

## 4. Discussion

As household finances due to COVID-19 worsened, there was a tendency for adolescents to become more dependent on smartphones. The following groups are particularly vulnerable to smartphone dependency associated with household financial decline: adolescents with high risk of smartphone dependency, girls, adolescents without financial support. and adolescents with three or more bathrooms on the family affluence scale. Concerning the vulnerability of adolescents with three or more bathrooms and their own bedroom, having their own room could serve as an independent space where adolescents could immerse themselves and reduce parental interference. Moreover, a house with three or more bathrooms may be large in size, resulting in a greater physical distance between family members and decreased opportunity for interaction. Adolescents from wealthy families with limited communication may feel more anxious about the economic downturn caused by COVID-19. Adolescents sometimes reinforce their social status through the socioeconomic status of their parents and gain popularity by acquiring expensive clothes and items [28]. The economic downturn caused by a pandemic, therefore, threatens their social status and weakens their control over an uncertain future.

There is broad consensus that economic crises have detrimental effects on mental health, including depression, anxiety, and insomnia [29]. During the 2009 Greek economic crisis, adolescents also became addicted to the internet [30]. Smartphone dependency among adolescents is also strongly associated with mental health issues, such as depression, anxiety, and attention deficit hyperactivity disorder [31]. Greeks were 2.6 times more likely to suffer from depression in 2011 than in 2008 [32]. Nomophobia (no-mobile-phobia or disconnection syndrome) was chosen as “Word of the Year” in 2018 by the Cambridge Dictionary [33]. This was also the year of the worst stock shock in a decade, with the global stock market losing about $12.3 trillion in market capitalization [33].

Smartphone dependency is also likely to affect girls and adolescents without financial support such as for tuition, lunch, school uniforms, and textbooks from people or institutions. Girls often use smartphones for social reasons, such as social network services (SNS) and shopping, whereas boys often use them for entertainment, e.g., games and movies [34]. The Korean government informs citizens of natural and social disasters through a cell broadcast service (CBS) by text messages. Once a pandemic was declared in 2020, CBS increased by approximately 60 times compared to 2019, and the number of CBS messages related to infectious diseases was 47,720 [35]. In addition, disaster notification text messages and crisis reporting in media can cause social unrest. Studies have found that such social tension can provoke FoMO in girls who are susceptible to depression and anxiety [36,37]. Furthermore, FoMO may increase SNS use because individuals constantly want to know what other people are doing and discussing. Such social unrest could be exacerbated during the uncertain and catastrophic COVID-19 pandemic. Similarly, smartphone dependency among girls may increase with the rise of nomophobia, where a smartphone is always nearby to receive alerts [38].

When adolescents have no outside form of financial support, they and their families may feel especially hopeless. When subjective material well-being is insufficient, it would be difficult to obtain materials such as stylish clothes and leisure expenses to form a bond with peers and thus adolescents may be marginalized. In addition, family, wealth, and the possibility of choice are key factors in subjective well-being which is closely related to self-esteem. Therefore, adolescents without financial support are at risk of low self-esteem [39]. COVID-19 caused an economic crisis, and 42.3% of adolescents expressed decreased confidence in society 42.3% [40]. Adolescents with increased social anxiety may be more dependent on smartphones for escapism or pleasure since social anxiety often involves the avoidance of real-time relationships [12]. COVID-19 has caused school closures and interrupted education for more than 1.6 billion learners in more than 190 countries [41]. Physical distancing to prevent the spread of COVID-19 and other infectious diseases can further isolate adolescents from financial or social support and make them dependent on a digital society.

In the relationship between household financial decline due to COVID-19 and the smartphone dependency questionnaire, social conflicts due to smartphone use were remarkable. Family conflicts arising out of smartphone use were more likely to happen in low-income households, and they had increased in low-income families in 2020, compared with that in high-income families in 2017, a pre-COVID-19 period. Considering that previous studies found that family relationships had improved due to increased family interaction during the COVID-19 pandemic, there may still be discord in less affluent families [42,43]. Compared with the pre-COVID-19 period, the content with the highest increase in smartphone use by adolescents during the pandemic consisted of movies, TV, and other videos, with an increase of 15.3% [2]. Adolescents also tend to prefer stimuli that can lead to aggression and intemperance [44]. Furthermore, parents’ financial hardship can cause adolescents to feel ashamed or inferior, which can lead to parent–child conflicts [45]. Unexpected and unpredictable events such as COVID-19 can especially increase feelings of helplessness, thereby intensifying smartphone dependency and parent–child conflicts.

To the best of the authors’ knowledge, this is the first study to identify an association between household financial decline due to COVID-19 and smartphone dependency by using national survey data collected from adolescents. Data from random cluster sampling are sufficiently representative of Korean adolescents [46]. Participants were approximately 55,000 individuals, between the ages of 12 and 18.

The limitations of this study are as follows. First, the causality between the COVID-19 household financial decline and smartphone dependency is obscure. As this study used a cross-sectional design, it was difficult to determine causality [47]. However, by comparing the degree of smartphone dependency between 2020 and 2017, we could estimate the impact of household financial decline due to COVID-19 on smartphone dependency. Second, self-reports may be inaccurate. Adolescents may not know their exact family income and may have responded falsely to avoid being perceived as poor. However, we additionally described the estimates of the adolescents’ household income level using the family affluence scale. Third, a selection bias may exist because the participants were students in attendance. Students who were absent from school, those who experienced literacy disabilities, those who were enrolled in home-schooling, and exceptional adolescents were excluded. However, Korean middle school education is compulsory and the high school entrance rate is 99.7% as of 2020 [48,49], which means that the possibility of selection bias may be minimized.

## 5. Conclusions

Household financial decline due to COVID-19 is closely related to smartphone dependency in adolescents, especially among girls and those with no financial support. Adolescents are vulnerable to smartphone dependency when their houses have three or more bathrooms and when they have their own bedrooms. Following the 2020 COVID-19 outbreak, adolescents experienced more family conflicts due to smartphone use. Proper care is required for adolescents, whose households have experienced COVID-19-related financial decline, regarding their mental health.

## Figures and Tables

**Table 1:** General characteristics of the study population.

Variables	Smartphone Dependency						
	Total		Yes		No		*p*-Value
	*N*	%	*N*	%	*N*	%	
Total (*N* = 53,534)	53,534	100.0	13,404	25.0	40,130	75.0	
Household financial decline due to COVID-19							<0.0001
No financial decline	15,923	29.7	3187	20.0	12,736	80.0	
Mild financial decline	21,350	39.9	5614	26.3	15,736	73.7	
Moderate financial decline	13,189	24.6	3671	27.8	9518	72.2	
Severe financial decline	3072	5.7	932	30.3	2140	69.7	
Gender							<0.0001
Boys	27,687	51.7	5697	20.6	21,990	79.4	
Girls	25,847	48.3	7707	29.8	18,140	70.2	
School grade (mean: 15.0, SD: 1.7)							<0.0001
7th grade	9800	18.3	1866	19.0	7934	81.0	
8th grade	9331	17.4	2298	24.6	7033	75.4	
9th grade	9161	17.1	2519	27.5	6642	72.5	
10th grade	8687	16.2	2188	25.2	6499	74.8	
11th grade	8669	16.2	2414	27.8	6255	72.2	
12th grade	7886	14.7	2119	26.9	5767	73.1	
Household income							<0.0001
High	20,895	39.0	4758	22.8	16,137	77.2	
Mid	25,738	48.1	6575	25.5	19,163	74.5	
Low	6901	12.9	2071	30.0	4830	70.0	
Financial support							<0.0001
Yes	5322	9.9	1464	27.5	3858	72.5	
No	48,212	90.1	11,940	24.8	36,272	75.2	
Family status							0.0003
Living with family	51,048	95.4	12,859	25.2	38,189	74.8	
Living without family	2486	4.6	545	21.9	1941	78.1	
COVID-19 infected city							<0.0001
Low	8957	16.7	2341	26.1	6616	73.9	
Mid	19,568	36.6	4657	23.8	14,911	76.2	
High	25,009	46.7	6406	25.6	18,603	74.4	
Academic level							<0.0001
High	19,767	36.9	4184	21.2	15,583	78.8	
Mid	16,241	30.3	3824	23.5	12,417	76.5	
Low	17,526	32.7	5396	30.8	12,130	69.2	
Perceived stress							<0.0001
Less	11,647	21.8	1674	14.4	9973	85.6	
Much	41,887	78.2	11,730	28.0	30,157	72.0	
Practicing exercise							<0.0001
Yes	41,786	78.1	9510	22.8	32,276	77.2	
No	11,748	21.9	3894	33.1	7854	66.9	
Sleep duration (mean: 6.2, SD: 1.5)							<0.0001
≤7 h	45,660	85.3	12,139	26.6	33,521	73.4	
8–10 h	7833	14.6	1262	16.1	6571	83.9	
≥11 h	41	0.1	3	7.3	38	92.7	
Family affluence (mean: 7.1, SD: 2.0)							0.0024
High (≥10 points)	5630	10.5	1404	24.9	4226	75.1	
Mid (6–9 points)	37,299	69.7	9480	25.4	27,819	74.6	
Low (≤5 points)	10,605	19.8	2520	23.8	8085	76.2	
BMI							<0.0001
Under-, normal weight	41,324	77.2	10,566	25.6	30,758	74.4	
Overweight, obesity	12,210	22.8	2838	23.2	9372	76.8	

All variables were expressed as the number of participants and percentages, and their *p*-values were significant below 0.05.

**Table 2:** The results of subgroup analysis stratified by independent variables.

**Variables**	**Smartphone Dependency, Low-Risk Group**								
	**Household Financial Decline Due to COVID-19**								
	**Mild Financial Decline**			**Moderate Financial Decline**			**Severe Financial Decline**		
	**OR**	**95% CI**		**OR**	**95% CI**		**OR**	**95% CI**	
Gender									
Boys	1.35	(1.25	1.46)	1.28	(1.16	1.42)	1.25	(1.09	1.44)
Girls	1.37	(1.26	1.50)	1.57	(1.43	1.73)	1.80	(1.54	2.10)
Financial support									
Yes	1.24	(0.98	1.57)	1.08	(0.86	1.36)	1.32	(0.98	1.77)
No	1.36	(1.28	1.45)	1.46	(1.36	1.56)	1.48	(1.33	1.65)
**Variables**	**Smartphone Dependency, High-Risk Group**								
	**Household Financial Decline Due to COVID-19**								
	**Mild Financial Decline**			**Moderate Financial Decline**			**Severe Financial Decline**		
	**OR**	**95% CI**		**OR**	**95% CI**		**OR**	**95% CI**	
Gender									
Boys	0.97	(0.76	1.22)	1.01	(0.78	1.31)	2.09	(1.54	2.83)
Girls	1.22	(1.02	1.45)	1.40	(1.14	1.72)	3.11	(2.32	4.16)
Financial support									
Yes	0.76	(0.41	1.41)	1.00	(0.56	1.78)	2.01	(1.05	3.86)
No	1.14	(0.98	1.32)	1.23	(1.04	1.45)	2.56	(2.05	3.21)

The variables in the left column were analyzed for low-risk cases and high-risk cases of smartphone dependency according to the severity of household financial decline due to COVID-19. Reference group: No financial decline (household financial decline due to COVID-19).

**Table 3:** Results of interaction analysis stratified by year and household income.

Variables	Smartphone Dependency											
	Smartphone Use (>2 h/Day)			Family Conflict Due to Smart Phone Use			Social Conflicts Due to Smart Phone Use			Difficulty in Work Due to Smart Phone Use		
	OR	95% CI		OR	95% CI		OR	95% CI		OR	95% CI	
**Year**												
2017	1.00			1.00			1.00			1.00		
2020	1.85	(1.80	1.91)	0.95	(0.93	0.97)	1.09	(1.05	1.14)	0.91	(0.89	0.93)
**Household income**												
High	1.00			1.00			1.00			1.00		
Mid	1.06	(1.03	1.09)	0.94	(0.91	0.96)	0.87	(0.83	0.92)	0.96	(0.94	0.98)
Low	1.21	(1.17	1.26)	1.07	(1.03	1.10)	1.05	(0.98	1.12)	1.07	(1.04	1.10)
**Year × Household income**												
2017 × High	1.00			1.00			1.00			1.00		
2020 × Mid	1.02	(0.99	1.04)	0.98	(0.95	1.00)	1.00	(0.95	1.05)	0.97	(0.95	1.00)
2020 × Low	1.01	(0.97	1.05)	1.06	(1.03	1.10)	1.06	(0.99	1.13)	1.04	(1.01	1.07)

Some items of the smartphone dependency scale were analyzed by adjusting for before (2017) and after (2020) of COVID-19 pandemic and household income. In KYRBS, the smartphone dependency scale was partially surveyed in 2017 and fully surveyed in 2020. Reference group: ≤ 2 h/day (smartphone use), no (family/social conflict(s)/difficulty in work due to smartphone use).

## Data Availability

Publicly available datasets were analyzed in this study. This data can be found here: (https://kdca.go.kr/yhs, accessed on 26 September 2021).

## Supplementary Materials

The following supporting information can be downloaded at: https://www.mdpi.com/article/10.3390/ijerph19063303/s1, Table S1: Contents of reliability analysis of categorizing smartphone dependency scale according to each item.
