# Supplementary material for: Association between the Perceived Household Financial Decline Due to COVID-19 and Smartphone Dependency among Korean Adolescents
Source: Int J Environ Res Public Health. 2022 Mar 11;19(6):3303. doi: 10.3390/ijerph19063303 (PMC8951075; doi:10.3390/ijerph19063303)
Supplement: Supplementary file 1 [file ijerph-19-03303-s001.zip › ijerph-1579848-supplementary.pdf]

Supplementary Table S1. Contents of reliability analysis of categorizing smartphone dependency scale according to each item

Category	Items	Average variance extracted	Composite reliability	McDonald's Omega
Control failure	Fail to reduce usage time<br>Difficult to control usage time<br>Difficulty keeping time properly	0.773	0.891	0.938
Salience	Difficulty concentrating on other things<br>Continuous thinking of smartphone<br>Strong urge to use smartphone	0.572	0.8	0.854
Problematic outcomes	Health problems due to use<br>Severe family conflict due to use<br>Severe social conflict due to use<br>Difficulty in work due to use	0.478	0.776	0.779

Each item followed the type classification of the smartphone dependency scale developed by National Information Society Agency of Korea.
